# iSECRETE: Integrating Microfluidics and DNA Proximity Amplification for Synchronous Single‐Cell Activation and IFN‐γ Secretion Profiling

**DOI:** 10.1002/advs.202309920

**Published:** 2024-08-22

**Authors:** Ri Lu, Yan Shan Ang, Ka‐Wai Cheung, Kai Yun Quek, Wei‐Xiang Sin, Elizabeth Lee, Shir Lynn Lim, Lin‐Yue Lanry Yung, Michael E. Birnbaum, Jongyoon Han, Lih Feng Cheow, Kerwin Kwek Zeming

**Affiliations:** ^1^ Critical Analytics for Manufacturing of Personalised Medicine IRG Singapore‐MIT Alliance for Research and Technology Singapore 138602 Singapore; ^2^ Graduate School for Integrative Sciences and Engineering National University of Singapore Singapore 119077 Singapore; ^3^ Department of Chemical and Biomolecular Engineering National University of Singapore Singapore 117585 Singapore; ^4^ National University Health System National University Hospital Singapore 119228 Singapore; ^5^ Yong Loo Lin School of Medicine National University of Singapore Singapore 117597 Singapore; ^6^ Department of Biological Engineering Massachusetts Institute of Technology Cambridge MA 02139 USA; ^7^ Department of Electrical Engineering Massachusetts Institute of Technology Cambridge MA 02139 USA; ^8^ Department of Biomedical Engineering National University of Singapore Singapore 117583 Singapore

**Keywords:** CAR T cells, cytokines, deterministic lateral displacement, immune profiling, microfluidics

## Abstract

Cytokines, crucial in immune modulation, impact disease progression when their secretion is dysregulated. Existing methods for profiling cytokine secretion suffer from time‐consuming and labor‐intensive processes and often fail to capture the dynamic nature of immune responses. Here, iSECRETE, an integrated platform that enables synchronous cell activation, wash‐free, and target‐responsive protein detection for single‐cell IFN‐γ cytokine secretion analysis within 30 min at room temperature is presented. By incorporating a DNA proximity assay (DPA) into a multifunctional microfluidic system, one‐pot homogenous cytokine signal amplification, with a limit of detection of ≈50 secreted molecules per cell is achieved. iSECRETE can robustly handle various sample types that are shown. Two distinct immune activation assay modalities are demonstrated on iSECRETE. Finally, the detection of single‐cell IFN‐γ secretion as an activation hallmark of chimeric antigen receptor T cells within 6 h of exposure to cancer targets is shown. iSECRETE represents the fastest single‐cell sample‐to‐result cytokine secretion assay to date, providing a powerful tool for advancing the understanding of biological phenotypes, functions, and pathways under in vivo‐like conditions.

## Graphical abstract

iSECRETE combines the advantages of integrated microfluidics and isothermal DNA proximity assay for a one‐step homogeneous single‐cell cytokine release assay. The assay can analyze whole blood or undiluted cell culture samples and detect activation‐induced cytokine release in live immune cells. iSECRETE is performed on chimeric antigen receptor (CAR) T cells cytotoxicity assay showing CAR T functional cytokine secretion.

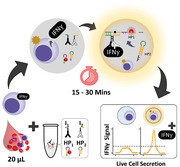

## 1 Introduction

Cytokines are powerful immune‐modulatory proteins responsible for immune functions such as inflammation, communication, differentiation, and immune effector functions. The dysregulation of cytokine secretion may exacerbate disease states such as sepsis and cytokine release syndrome. Innate and adaptive immune cells store cytokines in granules and rapidly release them in seconds upon activation.^[^ ^1^, ^2^, ^3^ ^]^ Existing commercial single‐cell cytokine assays based on transcriptomics, gene expression, and intracellular staining^[^ ^4^, ^5^, ^6^, ^7^ ^]^ do not measure the functional extracellular cytokine secretion and require a laborious processing time of 12 h to weeks.^[^ ^8^, ^9^, ^10^ ^]^ Developments of microfluidic single‐cell cytokine profiling methods aim to address these limitations. However, most microfluidic approaches still involve manual sample processing steps, and lengthy incubation time and exhibit lowered sensitivity of detection compared to existing ELISA methods.^[^ ^11^, ^12^ ^]^ These methods are incapable of measuring the dynamically changing immune cytokine secretion response which occurs in minutes. Therefore, a rapid assay capable of profiling actual cytokine secretion at the single cell level can enhance the understanding of how immune cells coordinate an inflammatory response, in turn allowing us to better phenotype patients, decipher disease pathogenesis, and discover potential therapeutic targets.

Intracellular cytokine staining (ICS)^[^ ^13^, ^14^, ^15^, ^16^ ^]^ and enzyme‐linked immunospot (ELIspot) assays^[^ ^7^ ^]^ are commonly adopted across laboratories to profile cytokine levels of single cells. The next generation of single‐cell multiplexed cytokine secretions uses micro‐well‐based assays such as the Beacon and Isoplexis systems.^[^ ^11^, ^17^ ^]^ While these new technologies enable single‐cell cytokine multiplexed profiling, a major limitation of these techniques often not discussed is the sample preparation step required for such complex assays. The sample preparation may span 6 to 12 h including chemical activation, red blood cell lysis, multiple centrifuge steps, and cell staining steps.^[^ ^9^, ^11^, ^17^ ^]^ Since sample preparation procedures are time‐gapped from cell measurement modality, the single‐cell profiling is, hence, asynchronous and decoupled from triggers or activation events. It is difficult to unequivocally exclude the possibility of cellular cross‐talks during these preparation processes to truly decipher the native immune response. Moreover, these methods are limited in throughput (100–10 000 cells/day), are costly, and are often developed as a laboratory process optimization tool. Thus, a large technological and clinical diagnostic need exists for rapid single‐cell cytokine profiling of immune cells to elucidate temporal dynamics of immune response.^[^ ^4^, ^18^ ^]^

The main complexity involved in sample preparation is the need for multiple reagent exposure and cell washing to prevent non‐specific signal amplification during cytokine profiling assays. Newer commercial solutions have designed “one‐pot” single reagent homogenous assays such as time‐resolved fluorescence resonance energy transfer (TR‐FRET) based technologies which support wash‐free cytokine detection allowing the potential to measure direct cytokine secretions of cells synchronous from cell activation events. These time‐resolved assays need to measure single‐cell signals in a static state for up to milliseconds for clear contrast, and one fluorophore signal is generated per cytokine molecule target. An inefficient energy transfer mechanism often leads to low signal count, limiting the analytical throughput and requiring sensitive and expensive instrumentation. Single‐step cytokine detection and signal amplification can be achieved by incorporating DNA tags, as previously demonstrated in a plug‐and‐play split proximity circuit design.^[^ ^19^, ^20^, ^21^, ^22^ ^]^ This method, also known as DNA proximity assay (DPA), has been robustly tested in bulk cell cultures and media samples but has not been adapted for single‐cell cytokine secretion assays.

Herein, we introduce an integrated platform for wash‐free, target‐responsive protein detection probing single leukocyte (from various biological samples) secretion within 15–30 min at room temperature. Termed integrated Single‐cell Encapsulation and Cytokine RElease TEst (iSECRETE), our platform enabled DPA for single‐cell cytokine secretion through a custom high‐throughput droplet cytometry system directly from raw samples to single‐cell secretion analysis. Specifically, the heart of the system is a customized microfluidic device that integrates cell extraction, washing, reagent priming, single‐cell droplet isolation, incubation, and droplet fluorescence readout in continuous flow. We first calibrated the sensitivity and dynamic range of the DPA assay with a limit of detection of 8 pM, ≈50 secreted interferon‐gamma (IFN‐γ) molecules per cell. Subsequently, we showed 2 distinct iSECRETE assay modalities, an ex vivo activation test and an isolated single‐cell synchronous activation test, which can be easily configured through our integrated microfluidic platform. We validated that the iSECRETE assay is applicable to a wide range of samples, including whole blood and suspension‐cultured cells. Lastly, we demonstrated the detection of single‐cell IFN‐γ secretion as an activation hallmark of chimeric antigen receptor (CAR) T cell activation by B cell precursor leukemia cell line detecting IFN‐γ within 6 h of exposure to cancer targets.

iSECRETE, to our knowledge, is the fastest single‐cell sample‐to‐result cytokine secretion assay which has a huge impact on studying the live and dynamic nature of immune cell response, opening new opportunities to elucidate biological phenotypes, functions, and pathways closer to ex vivo conditions.

## 2 Results

### 2.1 Homogenous DPA Reaction Detects IFN‐γ in 10pL Droplet

**Figure 1:**
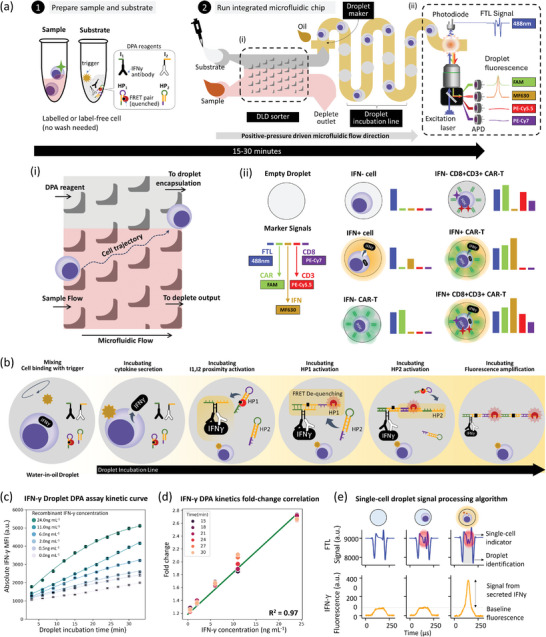
Schematics of the iSECRETE protocol which includes the reaction of homogenous DPA reaction for IFN‐γ and the microfluidic chip. a) schematics for droplet DPA. Droplet DPA is a homogenous reaction for which the binding of IFN‐γ cytokine target initiates a proximity‐triggered hybridization chain reaction and generates a turn‐on fluorescence signal. b) iSECRETE assay schematic. The microfluidic chip schematics for one‐step cell wash, extraction, DPA mix, and isolating single‐cell reaction within droplets using deterministic lateral displacement technology. A 5‐min run of whole blood results in close to 10 000 WBC cells encapsulated in droplets for cytokine secretion detection. The cytokine secretion can be detected within 15–30 min of room temperature incubation. c) DPA kinetic curve recorded by reacting human recombinant IFN‐γ to DPA reactants in a droplet. d) DPA standard curve measured at droplet incubation time from 15 to 30 min. Fold change reflects the ratio between the signal of a droplet containing IFN‐γ and DPA reactants and a droplet containing DPA reactants only. e) Schematics of three types of droplets seen here for FTL and DPA fluorescence signal. The FTL signal indicates an empty droplet with a background DPA signal, a droplet with a cell without a DPA signal, and finally a droplet with a cell and DPA peak signal indicating the presence of IFN‐γ. FTL: Front transmitted light; PD: Photodiode; APD: Avalanche Photodiode.

We developed room temperature droplet DPA assay as a rapid and sensitive method to profile secreted cytokine from a single live cell (**Figure** **1**a). DPA assay has 4 components. Oligonucleotide initiators anti‐IFN‐γ antibody‐oligo‐1 (I1) and anti‐IFN‐γ antibody‐oligo‐1 (I2) are each conjugated with one of the matched antibody pairs, for specific target recognition. Hairpin‐1 (HP1) and Hairpin‐2 (HP2) are a pair of DNA hairpin monomers that are opened in a cascaded manner during hybridization chain reaction (HCR) for signal amplification. HP1 is modified with a fluorescence reporter (mFluor630) and a corresponding FRET quencher such that they are next to one another in the closed hairpin state. The binding of I1 and I2 to cytokine targets brings oligonucleotide initiators in proximity, which then initiates HCR between HP1 and HP2 (Figure 1b; Figure S1, Supporting Information). The cascaded opening of HP1 generates a turn‐on fluorescence signal as the fluorophore and quencher are now separated in the opened hairpin state. This immunoassay detection method is a one‐pot reaction that, unlike the gold standard ELISA, does not require any sequential washing and addition steps. Compared with the proximity extension assay (PEA), which similarly utilizes the proximity‐activation of antibody‐oligonucleotide probes, DPA is enzyme‐free and operates isothermally (at room temperature in this work), which enables the study of a live cell.

IFN‐γ, a major cytokine during inflammation, was identified as the target cytokine of interest in this work. We optimized HP1 and HP2 concentrations for a room‐temperature droplet assay, and a 200 nM concentration showed the maximum signal‐to‐noise ratio (see Figure S2, Supporting Information). To obtain a reference value for single‐cell IFN‐γ concentration per droplet, we characterized droplet DPA assay using human recombinant IFN‐γ at different concentrations from 0.5–24 ng mL^−1^. The fluorescence signal of the various IFN‐γ concentrations increased from the 6‐min mark relative to the background control signal (Figure 1c). The assay time at 15–30 min showed a consistent fold change increase in fluorescence levels for the current dynamic range tested (Figure 1d). The reaction does not plateau and remains linear at 30 min suggesting continued signal amplification and an ideal linear range for detection. The limits of detection (LOD), defined as 3 standard deviations above background signal, at 15–30 min assay time was calculated to be ≈0.13 ng mL^−1^. It is important to note that at this concentration, the absolute IFN‐γ amount in a single droplet is around 50 molecules, given that the volume of each droplet is ≈10 pL. This is unprecedented given the readout time and the sensitivity of the homogenous DPA reaction coupled within a droplet assay.

### 2.2 Integrated Microfluidic and Custom Cytometry Setup for Single Cell Droplet DPA Assay

For droplet DPA to profile single cells from complex biological samples, a complementary iSECRETE platform comprising of an integrated microfluidic chip and a customized cytometry sampling system was developed. Figure 1a shows the schematics of the microfluidic chip based on deterministic lateral displacement (DLD) technology for the effective isolation of immune cells from a suspended biological sample ranging from cell cultures to whole blood (see Material and Methods).^[^ ^23^ ^]^ The DLD technology is a continuous‐flow precision cell sorting technology that has been shown to effectively perform sorting, washing, and profiling cells in varying cell concentrations from 10^2^–10^9^ cells mL^−1^.^[^ ^23^, ^24^, ^25^ ^]^ A 400mBar driving pressure was sufficient to process and effectively sort of WBCs from whole blood samples with a throughput of 4–10 million blood cells min^−1^ and an approximate nucleated cell count of 3000–5000 cells min^−1^. The leukocyte recovery and purity are consistently over 95% from whole blood as shown in our previous works.^[^ ^25^, ^26^, ^27^ ^]^ The final encapsulated droplets were of diameter 28 microns corresponding to 11pL of droplet volume. At this flowrate, the cells transit through the microfluidic device at an approximate velocity of 11 mm s^−1^ which would take ≈3 s to sample, wash, sort, mix, and finally be encapsulated in a droplet (see Figure 1a; Figure S3, Supporting Information). This rapid and almost negligible transit time will enable the cell to be captured into droplets with minimal perturbation and profiling them as native as possible.

A custom fluorescence cytometry setup was developed for high‐throughput measurement of droplet DPA signal. Droplets were profiled on the fly through a linear microfluidic channel of 20 µm width (see Figure S4, Supporting Information). A top photodiode (PD) was implemented to capture the front transmittance light (FTL) signal, which showed signatures of light interaction with a passing droplet containing a cell (Figure 1a). Up to 5 fluorescence wavelengths could be sampled from each droplet simultaneously by 5 avalanche photodiodes (APD). The DPA reporter for IFN‐γ used in this work fluoresces at 630 nm, which is illustrated as the orange signal line in Figure 1a. As a fully customized microfluidic and cytometry system, iSECRETE provides a robust and versatile process to measure single‐cell cytokine secretion. The following results will show different modes of using iSECRETE and flexibility for probing the IFN‐γ secretion and immune response of different samples with pre‐stimulation followed by DPA and/or co‐stimulation with DPA.

### 2.3 Label‐Free Ex Vivo Leukocyte IFN‐γ Secretion Assay

**Figure 2:**
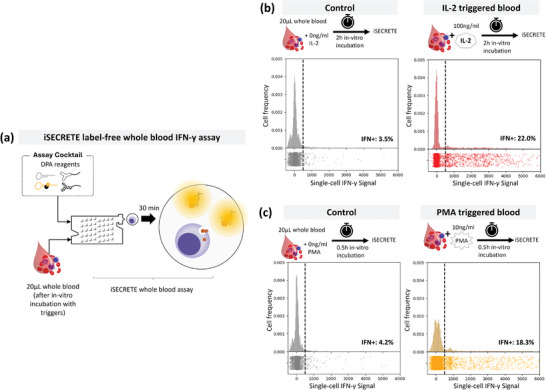
Label‐free immune cell IFN‐γ quantification from whole blood samples. a) The schematics of whole blood leukocyte iSECRETE IFN‐γ profiling without labeling of cells. 20 µL of blood is directly infused into the chip with a co‐flow of DPA reagent cocktail. b) Profiling of IFN‐γ secretion of circulating leukocytes within whole blood with controls and stimulated with Interleukin‐2 (IL‐2) and c) IFN‐γ secretion of blood cells triggered by phorbol 12‐myristate 13‐acetate (PMA). IL‐2 (100 ng mL^−1^) and PMA (10 ng mL^−1^) were added to 20 µL of whole blood prior to direct loading for iSECRETE IFN‐γ profiling. A threshold gating denoted in a dotted line was used to gate positive IFN‐γ secreting versus non‐secreting IFN‐γ leukocytes. The percentage of cells secreting IFN‐γ is denoted in the plot. N = 3150 total cells profiled for each group.

iSECRETE is a powerful platform that can directly process whole blood samples and quantify the rapid cytokine secretion of live single cells (**Figure** **2**). Label‐free identification of leukocyte‐containing droplets was achieved via an FTL signal (Figure 1e). The iSECRETE assay profiling of immune response in undiluted blood was completed in less than 40 min, inclusive of the loading of undiluted blood. The low IFN‐γ single‐cell secretion levels, <50 molecules per droplet, are comparable to that of the empty droplets (basal reading). True positive secretion of IFN‐γ was quantified by taking the difference between the peak absolute fluorescence reading and basal reading.

Interleukin‐2 (IL‐2) is commonly known to promote T‐cell growth and development;^[^ ^28^ ^]^ Phorbol 12‐myristate 13‐acetate (PMA) is a broad activator of immune cells, here used to simulate the rapid response of immune cells in a whole blood setting with just 0.5 h of trigger incubation time. We tested a total of 3 sample conditions – unstimulated whole blood, 100 ng mL^−1^ IL‐2 in whole blood for 2 h, and 10 ng mL^−1^ PMA in whole blood for 0.5 h. Only 6.3% of leukocytes from unstimulated whole blood showed a positive IFN‐γ signal. In contrast, IL‐2 spiked group and PMA spiked group increased the population of IFN‐γ secreting cells by 22% and 18.3% respectively as well as the IFN‐γ secretion level (Figure 2b,c).

The total workflow time for iSECRETE, including stimuli exposure (2 h for IL‐2 and 0.5 h for PMA) and single‐cell profiling (< 30 min), was ≈3 and 1 h for IL‐2 and PMA, respectively. PMA elicited a distinct response with both increases in frequency and IFN‐γ levels despite a brief 0.5‐h stimulation. Similar to existing streamlined whole blood activation tests,^[^ ^31^ ^]^ iSECRETE could account for complementary activation of the whole immune response like neutrophil‐lymphocyte communication and key serum proteins orchestrating the immune cascade.

### 2.4 Synchronous iSECRETE Assay Quantifies Single Cell IFN‐γ Secretion Profile

Bulk stimulation of immune cells in cultures results in significant crosstalk between cells. Isolating and studying the immune response to a specific trigger at a single‐cell level enables mechanistic studies of immune cell functions. Here we show a modality and use of iSECRETE via pairing stimulation assays with IFN‐γ secretion profiling of single cells within droplets for the same incubation time of 30 min without cell washing. For assays that require specific T‐cell stimulation, iSECRETE can be easily modified to add the immune stimulus in the homogenous DPA buffer within the droplet reaction. This enabled the synchronous study of trigger‐specific immune responses commonly used to measure the function of T‐cells.

**Figure 3:**
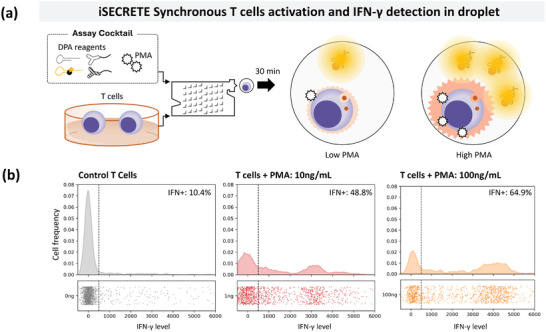
Applications of iSECRETE single‐cell stimulation assay a) Schematics for single‐cell stimulation assay using unspecific trigger PMA. T cells from the culture are washed and co‐encapsulated with PMA into single‐cell droplets. The fluorescence signal is sampled after 30 min of droplet incubation b) Histogram and scatter plot showing the distribution of IFN‐γ secretion profile, which shows T cell response to different PMA amount at a single‐cell level independent of cell‐cell interaction (*n* = 4046 for each group). The points to the right of the dotted line are classified as IFN‐γ^+^ cells.

CD3^+^ T‐cells were taken directly from the culture and infused into iSECRETE assay to be encapsulated in a mixture of PMA and homogenous DPA for IFN‐γ secretion detection for a synchronous assay (**Figure** **3**a). Similar to the whole blood assay, the cells in the droplet were identified in a label‐free manner using the FTL gating, and the cell‐positive droplets were profiled for IFN‐γ secretion. The frequency histogram plot can be seen in Figure 3b where the control or basal IFN‐γ secretion of T‐cells was gated by the dotted line. A reference gating is shown in a grey dot for the segregation of IFN‐γ secreting cell populations. In the presence of PMA co‐stimulation within the droplet, the Single‐cell IFN‐γ secretion level increases distinctly for 10 and 100 ng mL^−1^ PMA within a 30 min incubation and DPA reaction. Increasing levels of T‐cell IFN‐γ secretion were seen for PMA 10 ng mL^−1^ and PMA 100 ng mL^−1^ at 48.8% and 64.9%, respectively. The distinct shift in both the frequency histogram shows the viability of iSECRETE stimulation single‐cell assay for profiling and isolating the immune response from a single stimulant. It is possible to add inhibitors or additional co‐stimulants to study the effects on the cytokine secretion of immune cells. It is also viable to shorten the assay time, given the strength of stimulation and the sensitivity of the assay.

### 2.5 Labelled CD3/CD8 CAR T Cytotoxicity Assay

**Figure 4:**
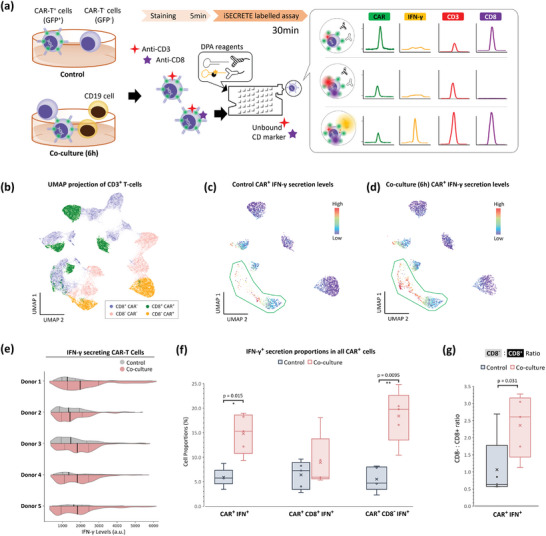
iSECRETE CD3 and CD8 phenotype profiling of chimeric antigen receptor T‐cells (CAR T) cytotoxicity with co‐culture of CD19 NALM6 target cells. a) Schematics of the experiment. Primary T cells were transduced by GFP‐CAR lentivirus and cultured for 14 days. The final culture contains both CAR^+^ cells (GFP^+^) and untransduced T cells (GFP^−^). An untreated control group and a 6‐h CD19 NALM6 target cell co‐culture group were prepared for 5 different T cell donors. 20 µL aliquot was taken from each sample group and stained with anti‐CD3‐PECy5.5 and anti‐CD8‐PECy7. Then, the stained sample was directly processed by iSECRETE microfluidics to wash away unbound CD markers and encapsulate single cells in droplets. Raw fluorescence data sampled from 3 different single CAR T cell droplets are shown in this graph. b) UMAP plot analysis for pooled CD3^+^ T cells) from control and co‐culture samples of 5 donors showing the clusters of various cell phenotypes. The UMAP plots showing IFN‐γ secretion levels of CAR^+^ cells in control and co‐cultured samples are shown in c and d, respectively. e) Donor‐level CAR T cell violin plot profile comparing control against co‐cultured. The solid black line marks the median single‐cell IFN‐γ secretion level, whereas the dotted line makes the 25th and 75th percentile. f) Paired plots show a significant increase in both percentages of IFN‐γ secreting cells for all CAR^+^, CAR^+^CD8^+^, and CAR^+^CD8^−^ cells. g) shows CD8^−^ to CD8^+^ ratio of control and co‐cultured IFN‐γ secreted CAR^+^ cells. All statistics were calculated based on a two‐tailed paired t‐test, with * and ** denoting *p* < 0.05 and *p *< 0.01, respectively.

The iSECRETE IFN‐γ platform can be easily adapted to screen for additional immune cell attributes such as cell surface protein expression. Four fluorescence channels were added to the customized cytometry platform with 488 nm laser excitation (See **Figure** **4**a; Figure S5, Supporting Information). CD19 CAR T cells were transduced via lentivirus and the CD19 CAR expression can be quantified by the co‐expressed GFP (525 nm) signal; IFN‐γ DPA reaction was detected at the same 630 nm channel; CD3 signals were detected at 690 nm which is critical to determine if the cell in the droplet is T‐cell or other cell types such as cancer cells; Lastly, CD8 fluorescence signal at 780 nm was used to further stratify the CD3^+^ T‐cells to CD8^+^ or CD8^−^ cells which are assumed to be CD4^+^ T‐cells. This multi‐channel and cell labeling modality of iSECRETE was tested with CAR T cells co‐cultured for 6 h with NALM6 cancer cells at a ratio of 1:2 to detect cytotoxic secretion of IFN‐γ during cancer cell targeting and killing. The co‐cultured samples with added surface marker fluorescence labels were directly loaded into the iSECRETE assay without any pre‐washing steps needed.

The iSECRETE profiling of CAR T specific IFN‐γ secretion assay was performed on 5 healthy donors, and the pooled single‐cell data was projected on an unsupervised UMAP clustering space with clear phenotypes of CD8 and CAR T cells shown in Figure 4b. The UMAP data of control (no exposure to NALM6 cells) and co‐cultured samples show a circled cluster of increased IFN‐γ secretion levels and numbers for CAR T exposed to NALM6 cells. The trends were observed in IFN‐γ secretion profiling in Figure 4c,d, with all donors showing an increase in IFN‐γ secretions for co‐cultured samples. This increase was also confirmed when compared with co‐culture negative control of non‐transduced cells (NTC) showing that CAR T IFN‐γ secretion is significant in CAR T cells compared to NTC (See Supporting Information SM1 and Figure S6, Supporting Information). We also compared this with an intracellular cytokine secretion (ICS) assay where similarly, the GFP CAR T CD19 targeting cells showed an increase in IFN‐γ secretion upon exposure to NALM6 cancer cells (Figure S7, Supporting Information). This shows iSECRETE can detect CAR T‐specific IFN‐γ secretion due to the presence of CD19 NALM6 target cells in co‐culture. There was evidence of donor‐to‐donor heterogeneity in both the IFN‐γ secretions as well as CD8+/‐ ratios in the control and co‐culture experiments (Figure 4e).

## 3 Discussion

In this study, we capitalize the integrated DLD‐to‐droplet microfluidic device to enable profiling live single‐cell cytokine secretion through a sensitive one‐pot immunoassay – iSECRETE. With a ≈3 s exposure from native sample, cell sorting, washing, reagent mixing, and single‐cell encapsulation, iSECRETE is advantageous over conventional bulk serum and ICS assays because it enables synchronous and live single‐cell functional snapshots, free from confounding factors such as cross‐stimulation and cell washing which inadvertently trigger cells. iSECRETE can also provide quantitative measurement of cytokines within 15–30 min at room temperature with a detection limit of ≈50 IFN‐γ molecules per cell, corresponding to 8 pM, which to our knowledge is an order of magnitude lower than existing single‐cell cytokine secretion methods (**Table** **1**). iSECRETE outperforms state‐of‐the‐art single‐cell cytokine profiling technologies listed in a comprehensive review by Platchek et al.^[^ ^30^ ^]^ Importantly, all existing cytokine profiling methods require pre‐analytical cell handling, isolation, and washing steps, which unintentionally perturb and alter leukocyte functionality.^[^ ^31^, ^32^ ^]^ This is especially critical when probing rapidly responding immune cells in whole blood. Taken together, iSECRETE could be a groundbreaking tool to enable sub‐hourly data integration for investigating dynamic cytotoxic events from immune cells, in line with the physiological timeframe of minutes.^[^ ^5^, ^33^ ^]^ Such utility was demonstrated by the whole blood test we established in this work.

**Table 1:** Comparison of single‐cell cytokine profiling state‐of‐art techniques. LOD: limit of detection.

	Process Whole Blood	Homogenous Assay	Total Assay Time	Cell Temporal Secretion	Detection Method	IFN‐γLOD	Minimum Incubation Volume
ICS^[^ ^35^, ^36^ ^]^	No	No	> 8 h	No	Antibody‐fluorophore	FACS machine dependent	Pipette dependent
ELISPOT^[^ ^37^, ^38^ ^]^	No	No	10–20 h	No	ELISA	–	384‐Well Plate
ISOPLEXIS^[^ ^11^ ^]^	No	No	24–48 h	Yes	ELISA	5 pM	500 pL
Dropmap^[^ ^12^ ^]^	No	Yes	5–6 h	Yes	Bead ELISA	200 pM	50 pL
iSECRETE	Yes	Yes	0.25–0.5 h	Yes	DPA	8 pM (50 molecules)	10 pL

A 30‐min assay duration was selected to maintain adequate contrast between highly active cells that secreted rapidly upon stimulation and slower secretors. We were able to stratify the cell population and identify highly active minority subpopulations of CAR‐T cells, potentially serving as a measure of CAR‐T efficacy. Additionally, the DPA assay continued to amplify until all HP2 molecules were reacted, reaching a maximal fluorescence signal. We designed the concentration of HP2 to be sufficiently high to provide a dynamic range suitable for IFN‐γ measurement. To ensure uniform incubation times across all droplets and comparable assay results, we adopted a fully integrated, continuous‐flow system for single‐cell secretion profiling. The incubation time of droplets was controlled by the fixed length of the incubation tube and the constant droplet flow rate driven by positive pressure. Thanks to the control of droplet incubation time by integrated microfluidics, our profiling system could maintain a robust measurement dynamic range to accommodate the variability of IFN‐γ secretion levels among different biological samples, while keeping the processing time short enough for rapid cytokine analysis, such as in evaluating the severity of sepsis or acute heart complications. Importantly, users of our system had the flexibility to calibrate the integrated microfluidic system to match the assay incubation time requirements for different assay reagents by varying the length of the droplet incubation tubing.

We demonstrated an in‐blood and a synchronous in‐droplet test model for capturing single‐cell heterogeneous cytokine release response exposed to various PMA concentrations. We also harness the customizability of microfluidic technology, injecting biochemical triggers through the assay cocktail inlet (Figure 1a and Figure 3a) to achieve a single‐cell level activation profile. The diffusion‐limited flow profile of the DLD device^[^ ^39^ ^]^ prevents the trigger interferences contributed by background from biological samples (red blood cells, platelets, and serum), maximally preserving the heterogeneous response of individual cells to triggers. A potential application of iSECRETE is to enable rapid high‐throughput screening of cell antigen‐antibody binding and their activation or inhibiting effects on specific immune cell populations.

Given that iSECRETE is an integrated microfluidic assay capable of directly handling whole undiluted blood samples, it has a unique advantage over current state‐of‐the‐art methods of providing granular information on systemic inflammatory secretion profiles and heterogeneity. Monocytes are known to produce IFN‐γ via CD14‐mediated activation^[^ ^39^, ^40^ ^]^ and human neutrophils are able to secrete, albeit unconventionally, IFN‐γ upon exposure to interleukins.^[^ ^41^ ^]^ The large proportions of IFN‐γ secretions in whole blood assay upon exposure to IL‐2 and PMA show an ex vivo nature of systemic immune response upon exposure to trigger(s). There is currently no tool available to profile functional cytokine secretion of the myriad subpopulations (e.g. granulocyte, monocytes, and lymphocytes) of whole blood leukocytes in such a rapid manner.

We further demonstrated the sensitivity and robustness of our assay by probing CAR T cells with their biological trigger (NALM6 CD19^+^ cells). Conventional assay for CD19 CAR T cells such as ICS requires cell fixing and long incubation time with target cells and secretion inhibitor to make statistically sound observations with the assumption that IFN‐γ cytokine produced in the cell will be secreted.^[^ ^43^ ^]^ In contrast, our method consistently detected a significant increase of IFN‐γ secreted from live CAR T populations with exposure to target NALM6 cells in co‐culture.^[^ ^43^, ^44^ ^]^ With the in‐droplet iSECRETE immune response test, it is interesting to see if iSECRETE can enable rapid CAR T cell potency and efficacy tests using solute‐based CD19 binding antibodies. We have shown that with a synchronous anti‐FMC63 CAR T stimulation in droplets containing isolated CAR T cells, an increase in IFN‐γ signals was measured with increasing anti‐FMC63 antibody (see Figure S8, Supporting Information). This method eliminates any potential crosstalk and measures the potency of each CAR T cell in a controlled environment. Additionally, an in‐droplet synchronous CAR T activation assay can rapidly profile the potency of CAR T cells without the need for operator‐dependent cell‐cell binding assay and decrease CAR T release test turnaround time to deploy time‐critical cell therapy products.

Given that many cell labeling and cell processing techniques like centrifuge are performed at varying temperatures, we wanted to minimize the effects of temperature gradient variation in such a rapid assay (15–30 min). Moreover, blood collected in EDTA tubes is transported within 2 h at room temperature. The DPA single‐cell secretion assay can be adapted for longitudinal studies at 37 °C in future studies. While at this temperature the cell secretions might be physiologically lower, the DPA assay is sufficiently sensitive to detect differences in basal immune cell activity.

Here we have shown a single protein target in iSECRETE as a preliminary proof‐of‐concept, and it is possible to integrate various custom DPA probes in multiple channels in the future. iSECRETE can also be a powerful tool when coupled with live cell enzyme secretion profiles as shown in Figure S9 (Supporting Information) where granzyme B is coupled with IFN‐γ profiles of CAR T cells. This opens possibilities for immune profiling. Given the highly customizable microfluidic setup of iSECRETE, it is possible for the technology to be deployed in a continuous flow manner for process control in cell manufacturing, biologics, and even viral production with modified DPA designs.^[^ ^45^ ^]^ Moreover, iSECRETE can handle whole blood (20 µL) to facilitate timely clinical diagnosis and continuous monitoring for CRS (cytokine release syndrome), which has a notable prevalence in cancer patients receiving CAR T cell therapy,^[^ ^46^ ^]^ as well as patients affected by infectious disease (e.g. COVID‐19)^[^ ^47^ ^]^ and autoimmune disorders.^[^ ^48^ ^]^

## 4 Experimental Section

### Integrated DLD Microfluidics for iSECRETE

iSECRETE uses an integrated microfluidic chip based on deterministic lateral displacement (DLD) L‐shape pillars for efficient isolation of immune cells. The chip design specification is based on previously validated designs with a critical cut‐off of D_c_ of 5.5 µm.^[^ ^27^ ^]^ This enables a sorting efficiency and recovery of >95% of WBCs. The sorted WBCs are simultaneously sorted and suspended in an enzyme FRET substrate medium to be encapsulated in water‐in‐oil droplets. The droplets were generated and incubated at room temperature for 30 min and reinjected into a standard cross junction for oil spacing between droplets to ensure consistent droplet spacing. Samples and buffers are contained in tube reservoirs which are subsequently injected into the device at a pressure of 400 mBar to ensure the effective sorting of immune cells from whole blood.^[^ ^26^ ^]^ Similarly, the microfluidic chips were made from polydimethylsiloxane (PDMS) with a master SU‐8 4‐inch wafer master mold fabricated with standard photolithography methods using a quartz chromed mask (JDphotodata, UK).^[^ ^26^ ^]^

### Optical and Detectors

iSECRETE uses a custom cytometry setup consisting of a continuous power 100 mW 485 nm Laser for excitation of fluorescence markers and 4 avalanche photodiode detector (APD) channels shown in Figure 1a. The laser excites the fluorophores through a 20 × 0.7 NA Olympus objective which also provides illumination for the CMOS camera (FLIR Blackfly S, US). The light is backpropagated into the objective and through mirrors and split into sequential low‐pass filters at wavelengths 525, 630, 690 nm, and a high‐pass filter at 780 nm (**Table** **2**). The corresponding fluorescence outputs were detected using Hamamatsu C12703 avalanche photodiodes (APD) (**Table** **3**). The voltage signals were converted in real‐time into bit quantization levels using 18bit DAQami (USB‐1808X) for downstream data processing. Cell detection was performed using a photodiode (Thorlabs) to measure light blocked by passing cells.

**Table 2:** Filter cut‐offs for fluorophore detection.

Filter type	Cutoff	Type	Fluorophore detected
Dichroic	525 nm	Low‐pass	FAM/GFP (to APD1)
Dichroic	630 nm	Low‐pass	mfluor630 (to APD2)
Dichroic	690 nm	Low‐pass	PE‐Cy5.5 (to APD3)
High‐pass	780 nm	High‐pass	PE‐Cy7 (to APD4)

**Table 3:** Biological markers detection in corresponding APDs.

APD channel	Fluorescence color	Fluorescence Probe	Biological Marker
APD‐1	Green	GFP	CAR Marker
APD‐2	Orange‐red	Mfluor630	IFN‐γ
APD‐3	Red	PE‐Cy5.5	CD3
APD‐4	Red‐Infrared	PE‐Cy7	CD8

### Homogenous DPA Reagents

All DNA oligonucleotides used in this study were purchased from Integrated DNA Technologies (IDT, Singapore, and US) with HPLC purification grade. The DNA sequences used in this study are shown in **Table** **4**:

**Table 4:** DNA sequences for HP1, HP2, I1, I2 were used in this study.

HP1	GTT GGA ATT GGG AG/iAmMC6T/ AAG GGC TCT TAC TTT GCC CT/iBHQ‐2dT/ACT CCC
HP2	GCCCTTACTCCCAATTCCAACGGGAGTAAGGGCAAAGTAAGA
I1	/5AmMC6/TTT TTT TTT TTT TTT GTG CCC TTC AAT TCC AAC AAG G
I2	GCCCTTACTCCGGGCACTTTTTTTTTTTTTTT/3AmMO/

The lyophilized DNA was reconstituted in 1× Tris–EDTA buffer (1× TE, pH 8.0) to give 100 µM stock and stored at 4 °C and protected from light.

A pair of purified mouse monoclonal antibodies (clone 4S.B3 and NIB42) against human Inf‐γ validated for ELISA application and recombinant human Inf‐γ (clone rh Inf‐γ) was purchased from Biolegend (US). The antibodies were concentrated to at least 1 mg mL^−1^, and buffer was exchanged to 1× phosphate‐buffered saline (pH 7.4) for conjugation reaction using an Amicron ultrafiltration column with a molecular weight cut‐off of 50 kDa.

The 4 DPA components HP1, HP2, I1, and I2 were diluted from stock solutions to aliquots of 125 uL for all experiments. The final concentration of HP1 and HP2 is 200nM each, and the final concentration of I1 and I2 is 20 nM each.

### Reagents

The following chemicals were used as received: sodium chloride (NaCl, ≥ 99.5%), magnesium chloride (MgCl2, ≥ 98%), sodium acetate, absolute ethanol, acetonitrile, dimethylformamide, and triethylamine were purchased from Sigma Aldrich. 1× TE (pH 8.0) was purchased from 1st BASE. Phosphate buffer was prepared using sodium phosphate monobasic dehydrate and sodium phosphate dibasic anhydrous purchased from Acros Organics and Fisher Scientific, respectively. mFluor Blue 630 succinimidyl esters were purchased from AAT Bioquest. microBCA assay was purchased from Thermo Fisher Scientific.

### Conjugation of a Fluorophore to HP1

Amine reactive mFluor Blue 630 succinimidyl esters were conjugated onto HP1 modified with an internal amine group and Black Hole Quencher 2. Briefly, 250 µM of HP1 was mixed with 2.5 mM of mFluor Blue 630 succinimidyl esters in 0.1m sodium bicarbonate (pH 8.3) reaction buffer. The conjugation reaction took place at room temperature for 60 min under vigorous thermomixer shaking (700 rpm). The dye‐HP1 conjugate was purified using ethanol precipitation. Briefly, 0.3 m sodium acetate (pH 5.2) was added to the conjugation product and mixed with 2.5 times the volume of absolute ethanol (stored at ‐20 °C). After 30 min incubation at −20 °C, the slurry was centrifuged at 14 000 rpm for 15 min at 4 °C. The pellet was washed twice with ice‐cold 70% ethanol and reconstituted in 1× TE buffer.

### Preparation of HCR Hairpin Working Solutions

The reaction buffer used in this study was 10 mM phosphate buffer (pH 7.4), 140 mM NaCl, 10 mM MgCl_2_, and 0.005% Tween‐20. Stock HP1 and HP2 (100 µM in 1× TE, pH 8.0) were diluted to 10 µM working concentration in the reaction buffer. They were heated to 95 °C for 5 min and snap‐cooled on ice for 30 min in separate tubes. For longer‐term storage of up to 3 months, the annealed oligonucleotides were kept in DNA LoBind tube (Eppendorf).

### Conjugation of an Oligonucleotide to Antibody

The initiator strands (I1 and I2) were modified with 5’‐ and 3’‐amine groups for conjugation with antibodies using a disuccinimidyl suberate (DSS) linker method.^[^ ^49^, ^50^ ^]^ with slight modification. Briefly, the oligonucleotide, DSS (dissolved in dimethylformamide), acetonitrile, and triethylamine were shaken at room temperature for 15 min, followed by ethanol precipitation as described in the method for HP1 conjugation. The activated oligonucleotide was reconstituted in 0.1 m sodium bicarbonate (pH 8.3) buffer, and a 3 molar equivalent amount was added immediately to the respective antibody solutions for incubation at room temperature for at least 2 h. The excess oligonucleotide was removed using an Amicon ultrafiltration column with a molecular weight cut‐off of 50 kDa and stored in 1× phosphate‐buffered saline (pH 7.4) at 4 °C until use. The antibody‐oligonucleotide concentration was quantified using a microBCA assay (for protein quantification) and by measuring the absorbance at 260 nm using Nanodrop (for oligonucleotide quantification).

### Blood Sampling Method

Venous whole blood samples were drawn at the National University Hospital Emergency Department and stored in 3 ml‐EDTA tubes with approval by the local Institutional Review Board (DSRB 2021/00246). Blood samples were kept at room temperature and processed within 2 h of phlebotomy.

### Stimulating Whole Blood with IL‐2

Two 50 µL aliquots (control group and test group) were sampled from EDTA whole blood (healthy donor) and kept in 200 µL Eppendorf tube. 5 µL of IL‐2 stock solution was added to the test group, to make the final IL‐2 concentration 100 ng mL^−1^. 5 µL of PBS was added to the control group. Both sample groups were incubated in 37 °C water bath for 2‐h before being processed by iSECRETE system.

### Stimulating Immune Cells with PMA

For blood experiments, PMA (Merck Sigma‐Aldrich, Singapore) was directly added into 20 µL of whole blood in a 500 µL Eppendorf tube and incubated in 37 °C water bath for 30 min. Stock solutions of PMA were added to the substrate solution at 10 ng mL^−1^ and 100 ng mL^−1^. CAR‐T cells were harvested from the culture medium and prepared as 50 µL of 100 000 cell mL^−1^ suspension for all test groups. The cell samples were directly processed by iSECRETE chip. The droplet incubation time was set to 30min.

### CD Marker Staining

First, the cells were gently resuspended in the culture well. 50 µL sample aliquot was taken for each individual experiment to be stained with a cocktail of 1.5 µL CD3‐PE Cy5.5 (Invitrogen) and 1.5 µL CD8‐PE Cy7 (Biolegend). After 10 min of staining at room temperature, the entire sample solution was loaded onto the microfluidic device for immediate processing.

### Experiment Setup

The integrated microfluidic device and the tubings were flushed with Pluronic F127 for at least 3 min and rinsed with 1x PBS prior to the loading of the cell sample and DPA reagents. Novec 7500 fluorinated oil was loaded to the droplet compartment of the device right before the initiation of flow by an automated pressure manifold system. In a total 40‐min continuous flow process, the T cells were sorted and washed from the original medium and then mixed and compartmentalized into single‐cell droplets containing just the DPA cocktails.

### Preparation of Lentivirus for Expressing Anti‐CD19 CAR in T Cells

A third‐generation lentiviral vector construct encoding a second‐generation 4‐1BB‐CD3‐zeta anti‐CD19 CAR with GFP expression downstream of an IRES (pHIV‐Myc‐CD19‐CAR‐4‐1BB‐CD3z‐IRES‐GFP), together with packaging plasmids pMDLg/pRRE (Addgene 12251), pRSV‐Rev (Addgene 12253), and pMD2.G (Addgene 12259), were used for preparing the lentivirus for anti‐CD19CAR expression in T cells. The lentivirus for anti‐CD19 CAR T cells (CD19CAR T cells) generation was produced using LV‐MAX Lentiviral Production System (ThermoFisher Scientific), followed by concentration of the lentiviral supernatant using Lenti‐X Concentrator (Takara Bio, Inc.) according to manufacturers’ instructions.

### CD19 CAR T Cell Generation

Peripheral blood mononuclear cells (PBMCs) were purchased from STEMCELL Technologies. To prepare the CD19 CAR T cells, frozen PBMCs were thawed, and the CD3^+^ T cells were isolated using the EasySep Human T Cell Isolation Kit (STEMCELL Technologies). The purity of the CD3^+^ T cells is > 95% as measured by flow cytometry. After isolation, the CD3^+^ T cells were activated using Dynabeads Human T‐expander CD3/CD28 (ThermoFisher Scientific) in the cell culture medium (AIM‐V (ThermoFisher Scientific) + 2% human serum (Merck) + 100U/ml human IL‐2 (Miltenyi Biotec)). Two days after activation, activated T cells were transduced with the lentivirus for anti‐CD19CAR expression, as mentioned above, at the multiplicity of infection (MOI) = 5. The lentivirus‐transduced CD19 CAR T cells were then expanded in AIM‐V+2% human serum+100U/ml human IL‐2 for another 12 days before use.

### Co‐Culture of CD19 CAR T Cells and NALM6 Cell Line

T cells were transduced with a CD19‐targeting CAR construct which co‐expresses green fluorescent protein (GFP) to identify transduced cells. Briefly, CAR T cells were prepared as mentioned above, and the IFN‐γ secretion of the CAR T was assessed by co‐culturing with NALM6 CD19 expressing B‐cell line at a ratio of 1:2. CAR T cell alone without any stimulation served as a negative control. For all co‐culture experiments, both the mixture of T‐cells and B‐cells are loaded onto the iSECRETE chip and thus, CD3^+^ labels are needed to identify T‐cell‐only droplets for IFN‐γ secretion profiling.

## Conflict of Interest

The authors declare no conflict of interest.

## Author Contributions

R.L. and Y.S.A. are the co‐authors. K.K.Z., Y.S.A., S.L.L., J.H. performed conceptualization. R.L., K.K.Z., Y.S.A., E.L., L.L.Y., K.C., W.S., K.Q., M.B., S.L.L. performed methodology. R.L., K.Q., K.K.Z., L.R., K.W.C., L.F.C., M.B. performed investigation. R.L., K.K.Z. performed visualization. K.K.Z., S.L.L., J.H. R.L. performed supervision, Y.S.A., K.K.Z. wrote the original draft. All authors wrote, reviewed and edited the original draft.

## Supporting information

Supporting Information

## Data Availability

The data that support the findings of this study are available from the corresponding author upon reasonable request.
