# Supplementary material for: iSECRETE: Integrating Microfluidics and DNA Proximity Amplification for Synchronous Single‐Cell Activation and IFN‐γ Secretion Profiling
Source: Adv Sci (Weinh). 2024 Aug 22;11(40):2309920. doi: 10.1002/advs.202309920 (PMC11516109; doi:10.1002/advs.202309920)
Supplement: Supplementary file 1 — Supporting Information [file ADVS-11-2309920-s001.docx]

Supplementary Data

iSECRETE: Integrating Microfluidics and DNA Proximity Amplification for Synchronous Single-Cell Activation and IFN-γ Secretion Profiling

*Ri Lu^1,2,#^, Yan Shan Ang^3,#^, Ka-Wai Cheung^1^, Kai Yun Quek^1^, Wei-Xiang Sin^1^, Elizabeth Lee^1^, Shir Lynn Lim^4,5^, Lin-Yue Lanry Yung ^3^ ,Michael Birnbaum^1,6^, Jongyoon Han^1,6,7,*^, Lih Feng Cheow^1,8,*,^ Kerwin Kwek Zeming^1,*^*

# co-first,

*co-coresponding

^1^ Singapore-MIT Alliance for Research and Technology, Critical Analytics for Manufacturing of Personalised Medicine IRG, Singapore

^2^ National University of Singapore, Graduate School for Integrative Sciences and Engineering

^3^ National University of Singapore, Department of Chemical and Biomolecular Engineering

^4^ National University Health System, National University Hospital

^5^ National University of Singapore, Yong Loo Lin School of Medicine

^6^ Massachusetts Institute of Technology, Department of Biological Engineering

^7^ Massachusetts Institute of Technology, Department of Electrical Engineering

^8^ National University of Singapore, Department of Biomedical Engineering

1. Supplementary methods
2. Supplementary figures
3. Supplementary methods

**Supplementary method SM1**

Interferon-gamma (IFN-γ) intracellular cytokine staining for flow cytometry

The GFP expression on CD19 CAR-T identified the T cells that are expressing the antiCD19 CAR after lentivirus transduction and hence can respond to the stimuli provided by the CD19 antigens on the CD19 expressing B-cell lines, NALM6. To induce the IFN-γ expression in the CAR-T cells, CD19 CAR-T cells were co-cultured with NALM6 CD19 expressing B-cell line at a ratio of 1 GFP+ T cells to 2 NALM6 cells for stimulation. For the intracellular cytokine staining of IFN-γ, CD19 CAR-T cells were co-cultured with NALM6 cells for 16 hours, BD golgiplug from the BD Cytofix/Cytoperm kit was added at the last 4 hours of the co-culture. After the co-culture finished, the cells were stained with LIVE/DEAD Fixable Aqua Dead Cell Stain Kit (ThermoFisher Scientific) followed by surface staining with CD3-AF700 antibody (eBioscience). The surface-stained cells were then fixed and permeabilized using the BD Cytofix/Cytoperm Plus (BD Biosciences). The fixed/permeabilized cells were stained with IFN-γ-BV605 antibody (Biolegend) followed by flow cytometry analysis using CytoFLEX S (Beckman Coulter). The IFN-γ expression in the GFP- (not expressing anti-CD19CAR) and GFP+ (anti-CD19CAR expressing) CD19 CAR-T cells were analyzed with FlowJo software (TreeStar).

1. Supplementary Figures


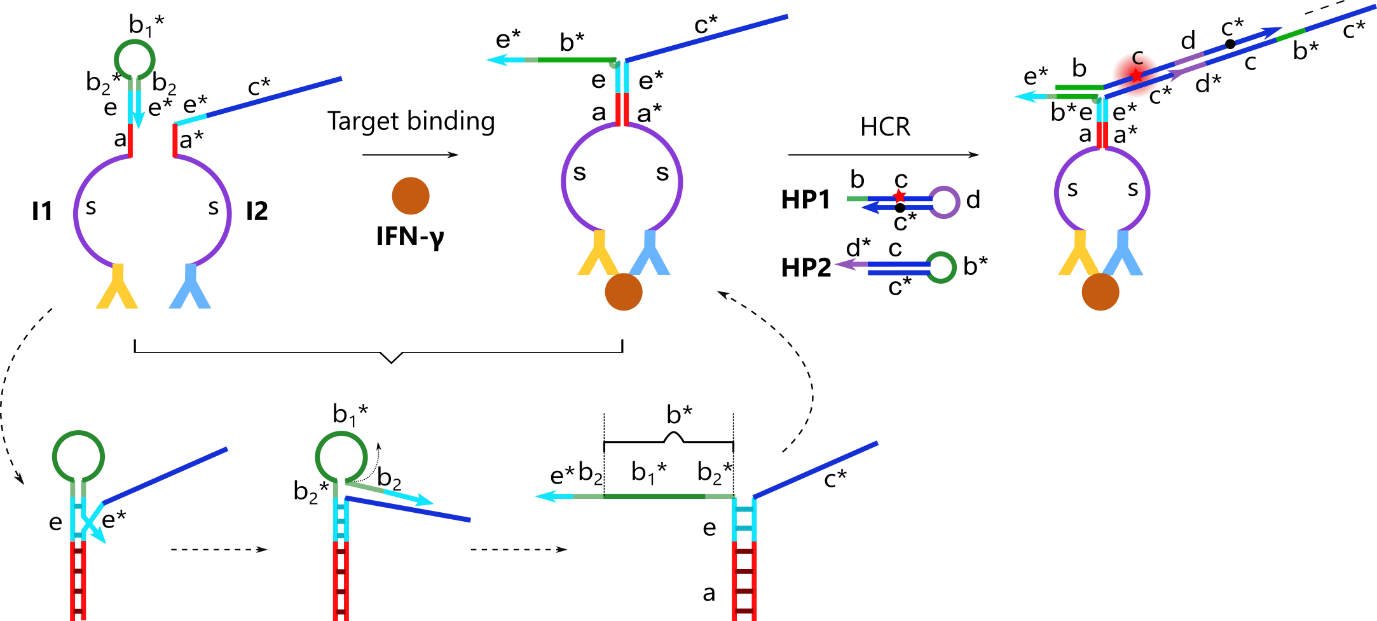


Supplementary Figure S1. Schematics depicting the steps involved in DNA proximity assay (DPA). The association region (domain a) on the initiator probes (antibody binders conjugated with I1 and I2) was designed to be short such that hybridization does not occur at RT. Upon target binding (IFN-γ), the increase in local probe concentration leads to proximity activation involving the sub-steps: association of domain a, displacement of domain e* in the hairpin structure and spontaneous dehybridization of domain b2. The complete trigger domains c* and b* initiators hybridization chain reaction (HCR) mediated by a pair of metastable hairpins (HP1 and HP2). The cascaded opening of HP1, facilitated by HP2, leads to a turn-on fluorescence signal due to the separation of the quencher (BHQ-2) from fluorophore (mFluor Blue 630).


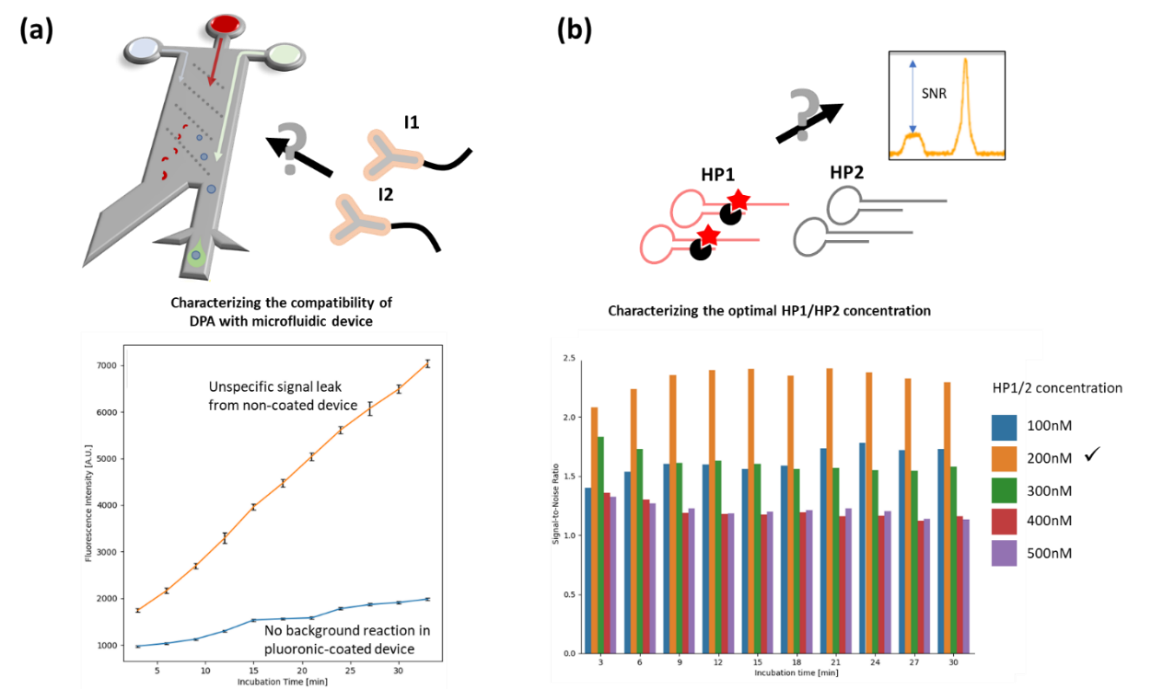


Supplementary Figure S2. Critical development to enable DPA on the integrated microfluidic platform. (a) shows the importance of Pluronic pre-coating of the microfluidic device in preventing unspecific background reaction triggered by the interaction between I1/2 to the active surface of the device. (b) shows that 200nM of HP1/HP2 is desired to give best signal-to-noise (SNR) ratio. As the trigger used in this experiment does not react all HP1/2, the SNR shown here is not the highest achievable SNR of the droplet DPA assay.

**Supplementary Figure S3. Calibration of transit velocity of cell in iSECRETE microfluidic device at different driving pressure.** Driving pressure for whole blood and T cell experiments is 400mbar. At this pressure, each cell can transit within 3s from inlet (in original biological sample) to droplet (in PBS with DPA reagents). PBS: phosphate buffer saline; DPA: DNA proximity assay

**
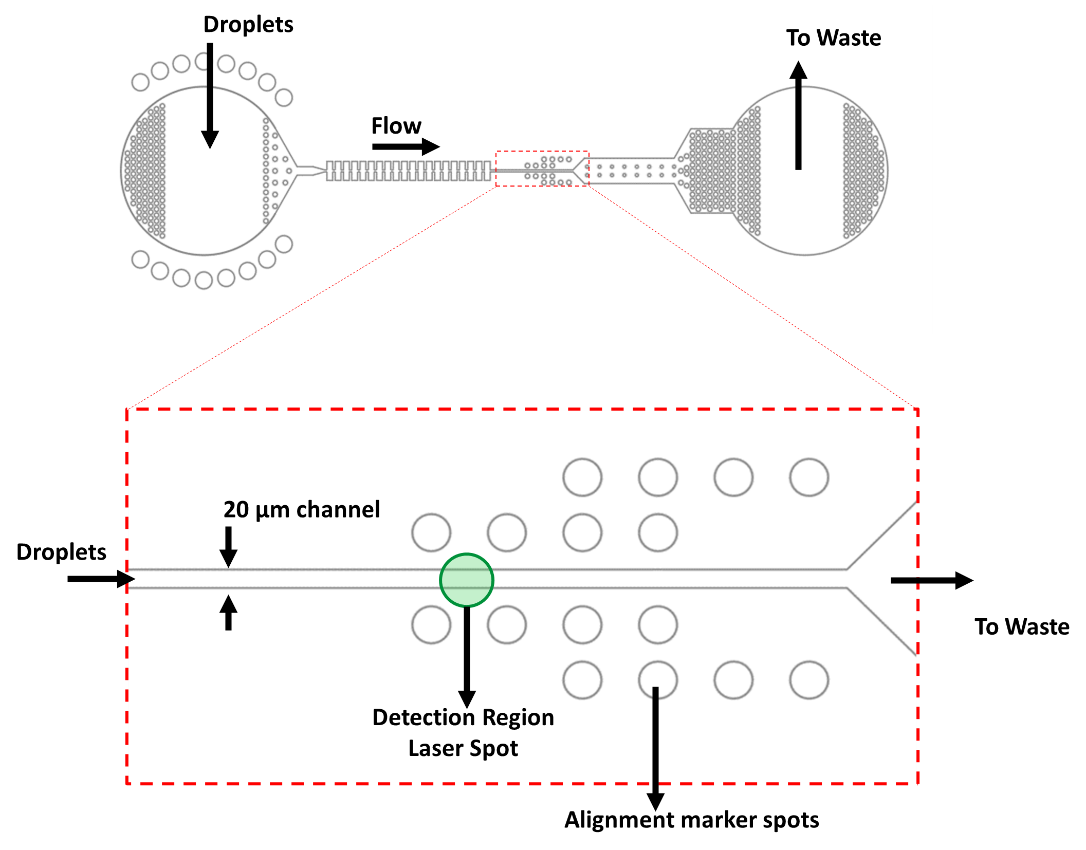
**

**Supplementary Figure S4.** The device schematics for the droplet cytometry observation window showing a 20 µm straight channel with alignment markers for detection region.


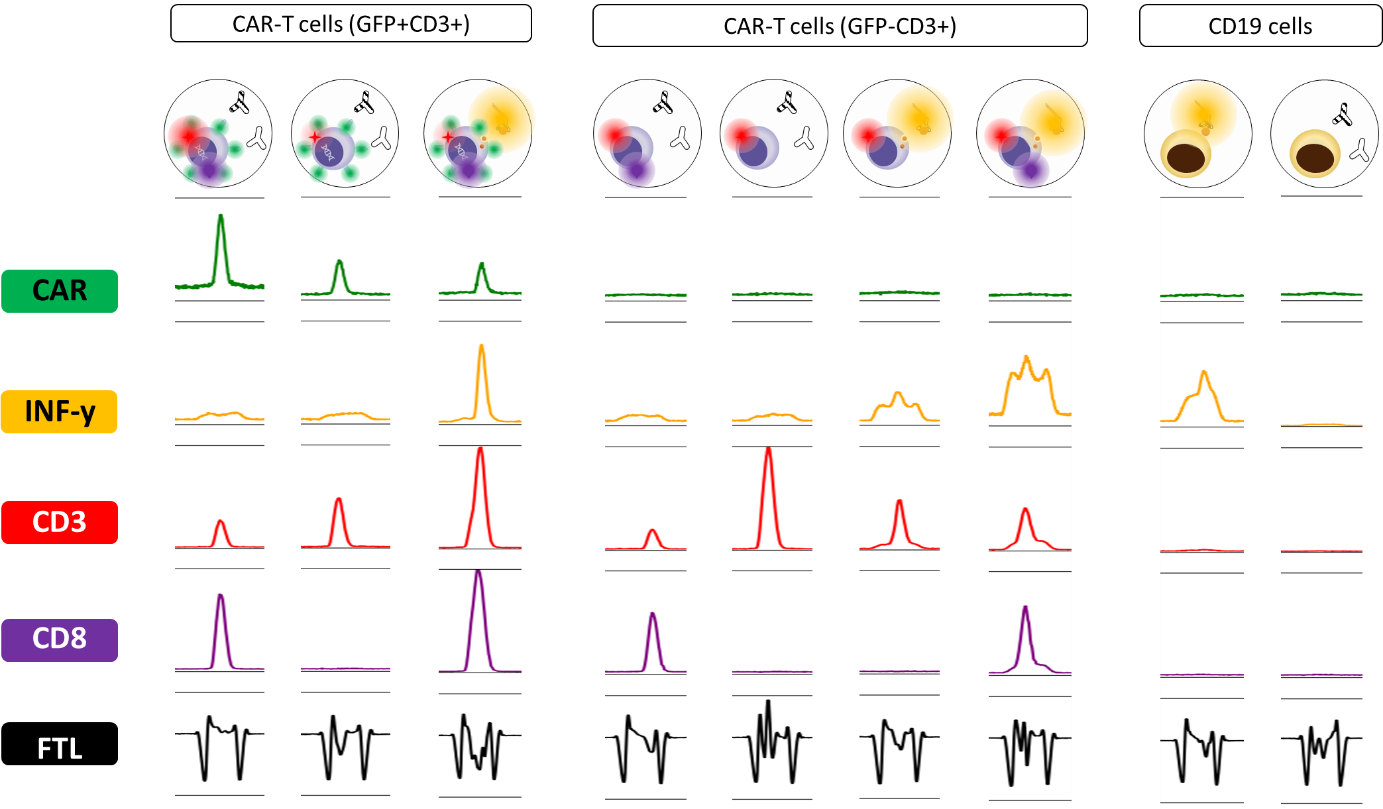


**Supplementary Figure S5.** **Raw fluorescence and front transmitted laser signal were measured in a CAR T co-culture experiment to distinguish various cell phenotypes**. The representative cell type was characterized by CAR expression (green, emitting at 525nm), IFN-γ secretion (orange, emitting at 630nm), CD3 (red, emitting at 671nm), and CD8 (purple, emitting at 778nm) surface marker expression. Quantification of secretion/expression relied on four corresponding fluorescence channel signals. The front transmitted light signal was utilized for detecting droplets containing cells. Abbreviations: CAR - chimeric antigen receptor, FTL - front transmitted light.


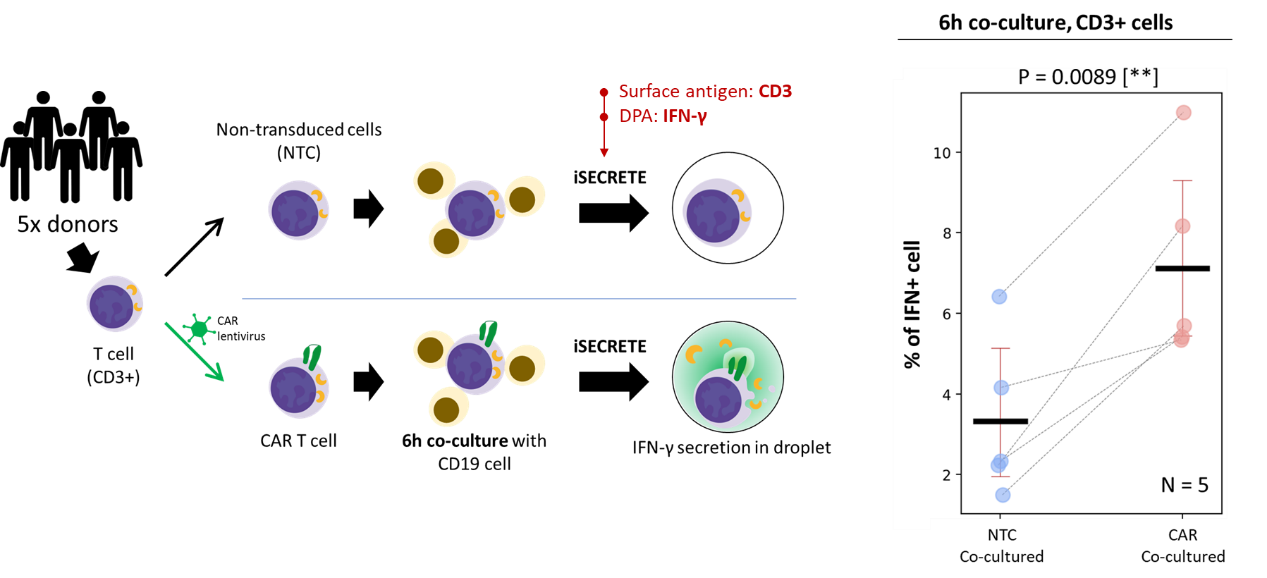


**Supplementary Figure S6.** IFN-y secretion levels of non-transduced cells (NTC) and CAR T cells were exposed to co-culture with CD19 cancer cells. 5 donor CD3+ T cells were isolated for this experiment. The proportion of %IFN-y secretion was significantly increased in CAR T cells compared to NTC cells upon 6hour exposure to CD19 cells. Two tailed paired students t-test was performed with ** denoting p-value < 0.01.


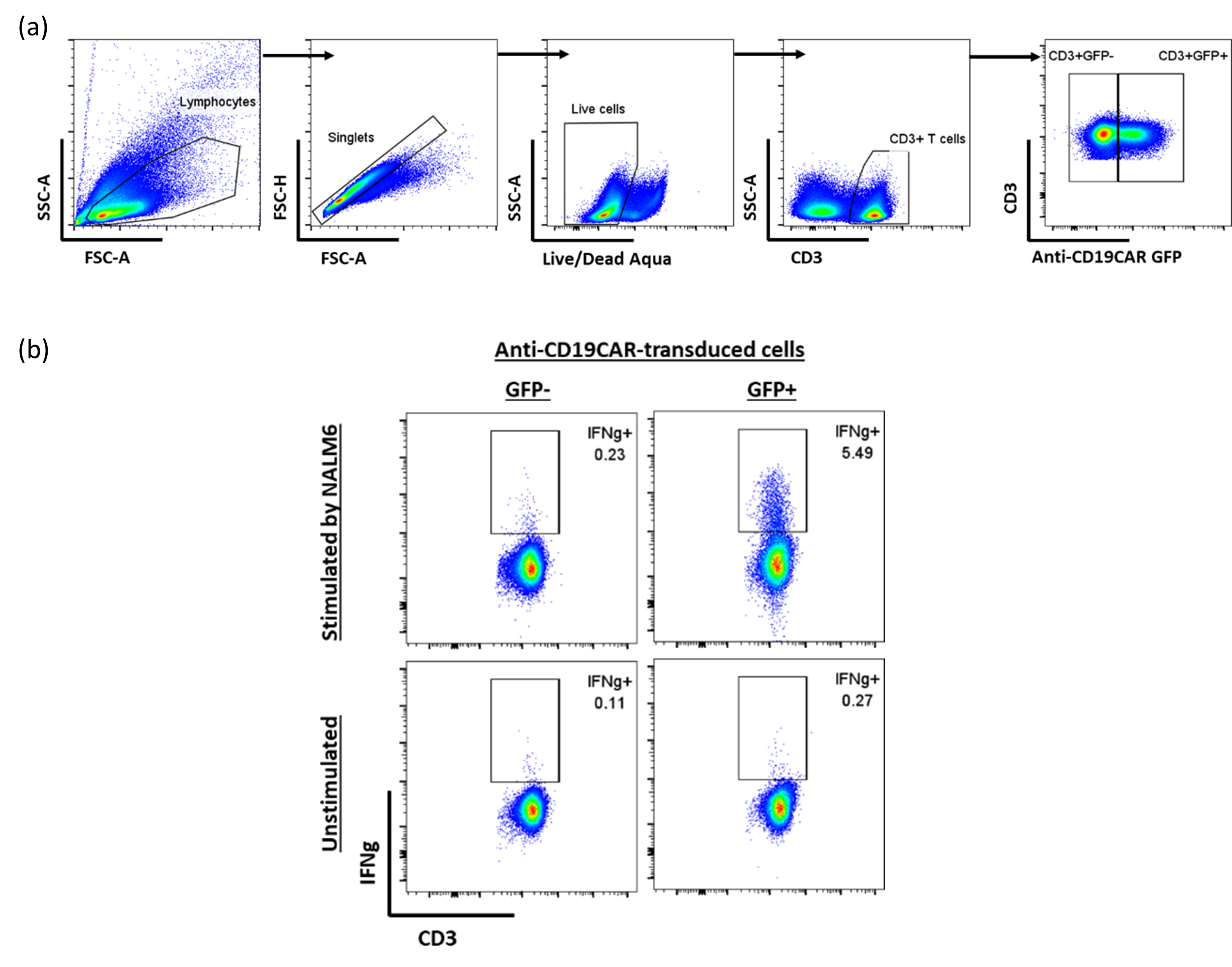


**Supplementary Figure S7. IFN-y Intracellular Cytokine Secretion (ICS) assay for the quantification of CD19 CAR-transduced Cells.** The FACS gating is shown in (a) with the gating of lymphocytes using FSC and SSC. Next, singlets were gated out to measure the amount of cytokine produced in each cell. The CD3+ T cells were selected for the IFN-y measurement. The final gating selects cells with CAR T expression and negative CAR T expressing cells. All populations were stimulated with NALM6 cancer cells and the data in (b) shows that stimulated GFP+ CAR T cells upon exposure to NALM6 cancer cells resulted in an increase of IFN-γ secretion.


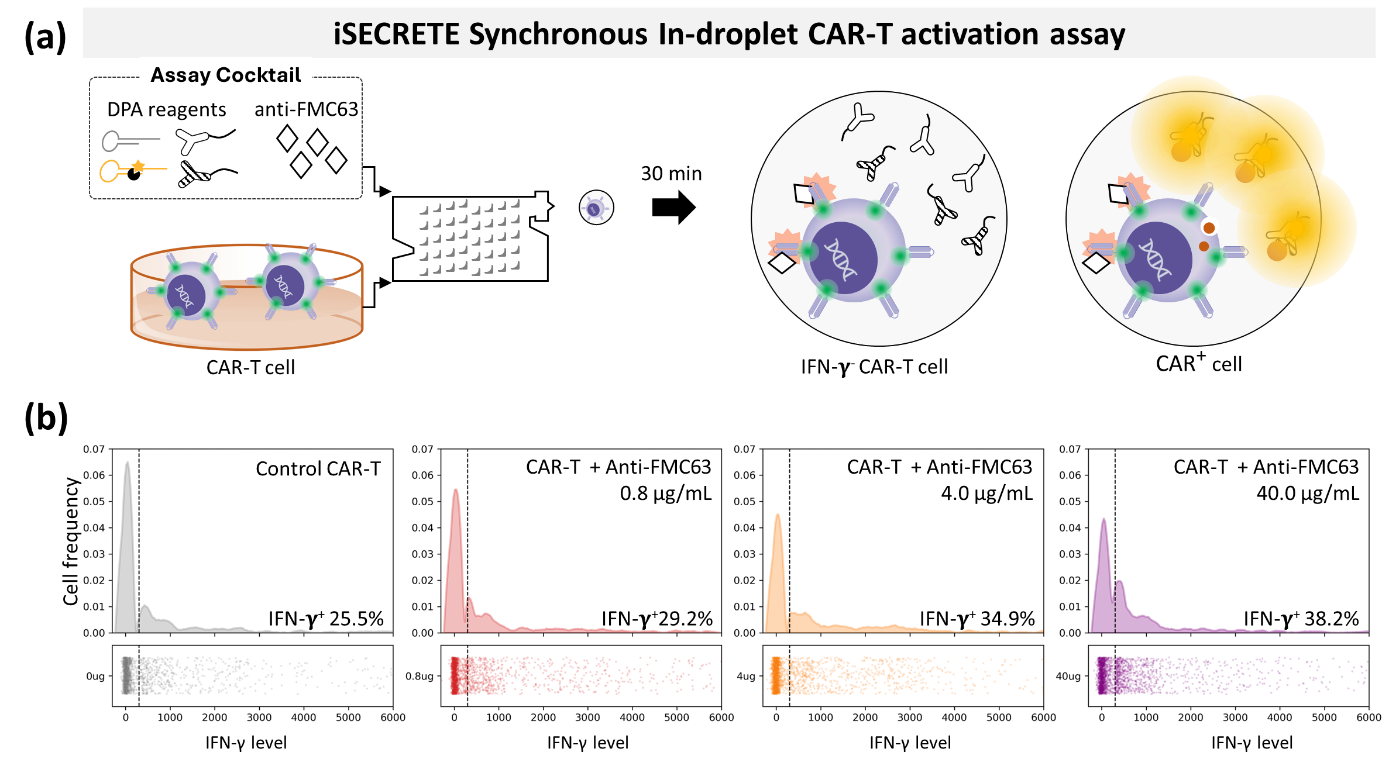


**Supplementary Figure S8. Testing single-cell isolated trigger on surface chimeric receptor from CAR T cell.** Peripheral blood T cells, sourced from a single donor, were tranduced into CAR T cells. T cells were stimulated and transduced with a lentivirus encoding the FMC63 chimeric antigen receptor (CAR). Post a 14-day culture period, we isolated 200,000 cells in 60µl. The CAR transduced cells was further subdivided into four independent aliquots of CAR T control, CAR T + Anti-FMC63 0.8, 4.0, 40.0 µg/mL respectively. The CAR T + Anti-FMC63 cohort underwent addition of soluble anti-CAR-FMC63-scFV antibody (acrobiosystems FM3-γ45). This experimental arrangement allowed for artificial stimulation in a meticulously isolated CAR T cell environment, eradicating background noise and cell-cell crosstalk. Preliminary observations indicated increase in the count of interferon (IFN) secreting CAR T cells and the magnitude of activation with the incremental FMC input. CAR: chimeric antigen receptor.


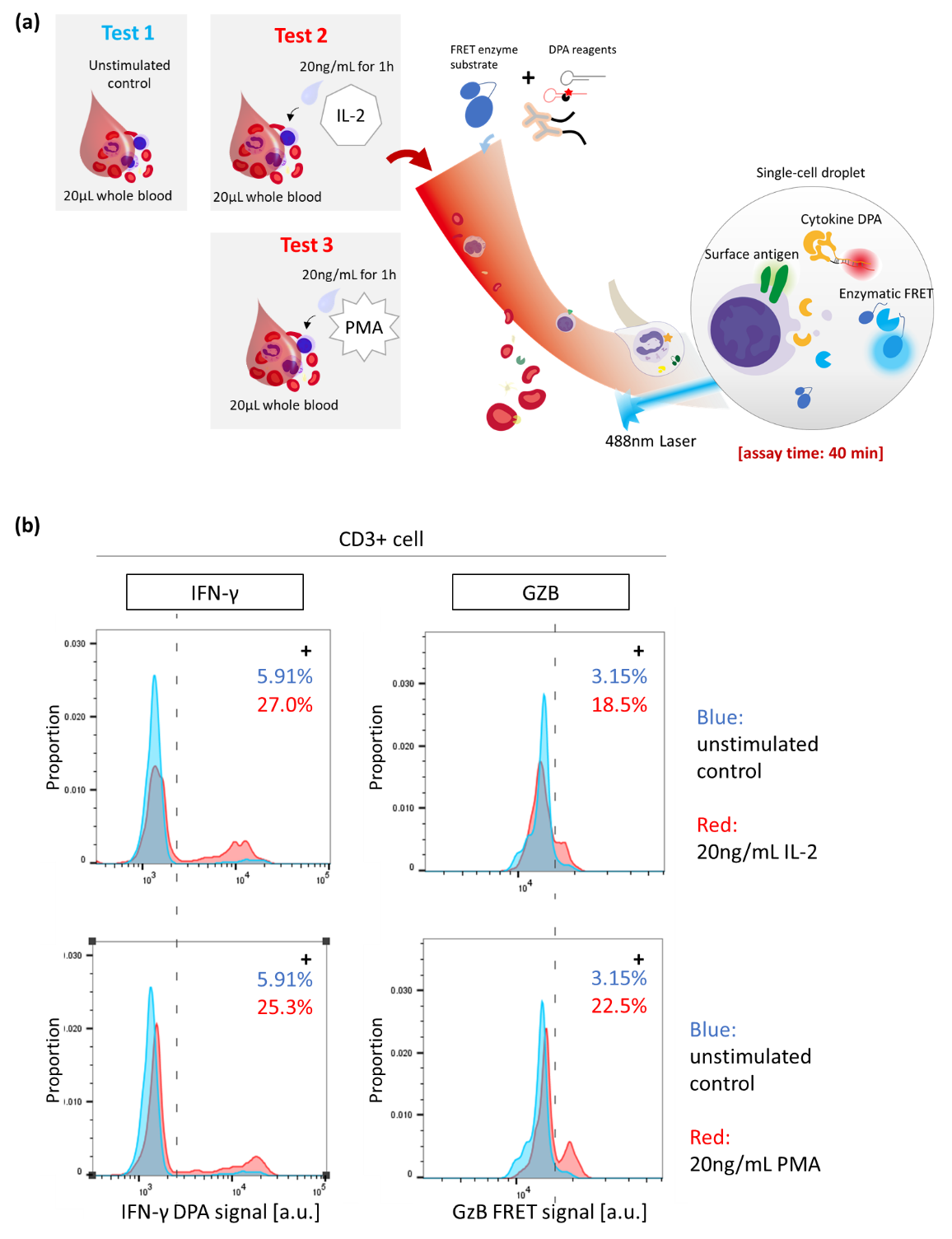


Supplementary Figure S9. Concurrent detection of single-cell enzyme and cytokine secretion. Whole blood from a healthy donor was aliquoted to 3x 20μL test groups. The conditions of the test groups are labelled in (a). A mixed panel of CD3 surface antigen staining, IFN-γ DPA reagents and Granzyme B (GzB) FRET substrate were mixed and used in this experiment. In a 40min assay, a significant increase in number of CD3+ WBC secreting GzB and IFN-γ is shown in (b), for both IL-2 and PMA stimulated group. This experiment is a proof-of-concept demonstration for the feasibility of combining all assays developed on our system.
